# Toward a Molecular Cytogenetic Map for Cultivated Sunflower (*Helianthus annuus* L.) by Landed BAC/BIBAC Clones

**DOI:** 10.1534/g3.112.004846

**Published:** 2013-01-01

**Authors:** Jiuhuan Feng, Zhao Liu, Xiwen Cai, Chao-Chien Jan

**Affiliations:** * Department of Plant Sciences, North Dakota State University, Fargo, North Dakota 58108-6050; † USDA-Agricultural Research Service, Northern Crop Science Laboratory, Fargo, North Dakota 58102

**Keywords:** BAC-FISH, cytogenetic map, RFLP genetic map, sunflower

## Abstract

Conventional karyotypes and various genetic linkage maps have been established in sunflower (*Helianthus annuus* L., 2n = 34). However, the relationship between linkage groups and individual chromosomes of sunflower remains unknown and has considerable relevance for the sunflower research community. Recently, a set of linkage group-specific bacterial /binary bacterial artificial chromosome (BAC/BIBAC) clones was identified from two complementary BAC and BIBAC libraries constructed for cultivated sunflower cv. HA89. In the present study, we used these linkage group-specific clones (∼100 kb in size) as probes to *in situ* hybridize to HA89 mitotic chromosomes at metaphase using the BAC- fluorescence *in situ* hybridization (FISH) technique. Because a characteristic of the sunflower genome is the abundance of repetitive DNA sequences, a high ratio of blocking DNA to probe DNA was applied to hybridization reactions to minimize the background noise. As a result, all sunflower chromosomes were anchored by one or two BAC/BIBAC clones with specific FISH signals. FISH analysis based on tandem repetitive sequences, such as rRNA genes, has been previously reported; however, the BAC-FISH technique developed here using restriction fragment length polymorphism (RFLP)−derived BAC/BIBAC clones as probes to apply genome-wide analysis is new for sunflower. As chromosome-specific cytogenetic markers, the selected BAC/BIBAC clones that encompass the 17 linkage groups provide a valuable tool for identifying sunflower cytogenetic stocks (such as trisomics) and tracking alien chromosomes in interspecific crosses. This work also demonstrates the potential of using a large-insert DNA library for the development of molecular cytogenetic resources.

The cultivated sunflower [*Helianthus annuus* L, x = 17, ca. 3000 Mbp/1C (Arumuganathan and Earle 1991)] is one of the leading edible oil crops worldwide. In the past decades, various genetic markers and the corresponding linkage maps have been developed, such as restriction fragment length polymorphism [RFLP (Berry *et al.* 1995, 1997; Gentzbittel *et al.* 1995, 1999; Jan *et al.* 1998)], simple sequence repeats [SSR (Gedil *et al.* 2001; Paniego *et al.* 2002; Tang *et al.* 2002, 2003; Yu *et al.* 2002, 2003)], expressed sequence tag (EST)-SSR, EST-INDEL (insertion or deletion), and EST−single-nucleotide polymorphism (Lai *et al.* 2005; Pashley *et al.* 2006; Heesacker *et al.* 2008)]. Moreover, the consensus genetic maps integrating the independently developed linkage maps based on the shared markers have been reported (Yu *et al.* 2003; Paniego *et al.* 2007). Yu *et al.* (2003) integrated 120 SSR loci from the public SSR map and 28 RFLP markers from Jan *et al.* (1998) map to a backbone of 80 RFLP loci on the genetic map of Berry *et al.* (1997). Another high-density composite genetic map (Paniego *et al.* 2007) was established by integrating a set of 161 SSR marker loci (Al-Chaarani *et al.* 2004) and 58 HA-SSR markers (Paniego *et al.* 2002) to the public SSR map (Tang *et al.* 2003). These maps with almost full sunflower genome coverage allow for the cross-reference with each other and provide a dense genome-wide framework for sunflower research.

Since the 1970s, cytogenetic studies of sunflower mostly focused on the karyotypic analyses using conventional Feulgen’s staining and C-banding (Raicu *et al.* 1976; Al-Allaf and Godward 1979; Cuellar *et al.* 1996; Kulshreshtha and Gupta 1981). In recent years, fluorescence *in situ* hybridization (FISH) has been used for chromosome characterization and karyotypic analysis using repetitive DNA sequences, such as rRNA gene, in sunflower [Table 1 (Schrader *et al.* 1997; Cuellar *et al.* 1999; Vanzela *et al.* 2002; De Paula Wilson *et al.* 2005; Ceccarelli *et al.* 2007)]. Although the results were sometimes controversial, the improvement of microscopic and cytogenetic techniques represents a significant step forward for the cytogenetic study in sunflower. The reasons for the inconsistent descriptions, such as the numbers of chromosomes with a secondary constriction, are mainly because of the high number, small size, and similar morphology of sunflower chromosomes. Therefore, chromosome identification and development of a universal karyotype is highly desirable in sunflower.

**Table 1:** Partial references reported on chromosome karyotypes and secondary constrictions in the genus *Helianthus* L.

	Chromosome Type			Secondary Constriction				
References	Meta-	Submeta-	Acro-	Satellite	45s rDNA	5s rDNA	Techniques and FISH Probes Used if Applicable	Materials
Raicu *et al.* 1976	10	3	4	3				
Al-Allaf *et al.* 1979	4	8	5	3				HI, HB, HR, HX
Cuellar *et al.* 1996	4	8	5	3				HA89, hybrid
Schrader *et al.* 1997	13		4	3	4	2	C-banding;	HA89
							45s rDNA: VER17	
							5s rDNA	
Cuellar *et al.* 1999	4	8	5*^a^*	3	3	2	C-banding;	*H. argophyllus*;
							45s rDNA: pTa71	*H. annuus*
							5s rDNA: 36pBG13	
Vanzela *et al.* 2002				2, 3, 4 (2x)	2, 3, 4 (2x)		C-banding	diploid (2×)
				4 (4x)	4 (4x)		C-CMA banding	tetraploid (4x)
				6 (6x)	6 (6x)		45s rDNA: pTa71	hexaploid (6x)
Ceccarelli *et al.* 2007	13		4	3	4		tandem repeats	HA89, RA20031, HOR
							45s rDNA: pTa71	
Talia *et al.* 2010	12	1	4*^a^*	3	3		repetitive retrotransposon-like sequences; homologous rDNA	HA89

Meta-, metacentric; Submeta-, submetacentric; Acro-, acrocentric or subtelocentric.

a Subtelocentric.

With the increasing availability of various molecular markers, the linkage maps are becoming more saturated in sunflower. Meanwhile, the impressive methodological and conceptual advances in molecular cytogenetics, such as bacterial artificial chromosome (BAC)−FISH, have received considerable attention for the studies of the plant genome (Zwick *et al.* 1998; Dong *et al.* 2000; Cheng *et al.* 2001; Kulikova *et al.* 2001; Islam-Faridi *et al.* 2002; Howell *et al.* 2002; Pedrosa *et al.* 2002; Kim *et al.* 2002, 2005; Stephens *et al.* 2004; Yoshido *et al.* 2005; Wang *et al.* 2007; Lamb *et al.* 2007; Fonsêca *et al.* 2010; Ohmido *et al.* 2010). In sunflower, *in situ* hybridization using a BAC clone containing highly repetitive retrotransposon-like sequences combined with rDNA sequence allowed the characterization of sunflower karyotype (Talia *et al.* 2010). The use of BAC clones containing single-copy sequence of SSR markers associated with agricultural important traits detected a FISH signal on specific chromosomes (Talia *et al.* 2011). Overall, however, the relationship between the linkage groups and individual chromosomes of sunflower remains unknown. The linkage groups have not been anchored to individual chromosomes due to the lack of proper genetic stocks or technical difficulties of chromosome identification in sunflower.

Alignment of the linkage groups with individual chromosomes has become possible with the recent development of efficient FISH techniques and the establishment of a BAC library and RFLP genetic map in sunflower. Previously, we developed an RFLP linkage map with 232 cDNA probes on 20 linkage groups [3−4 small linkage groups were not integrated with the others (Jan *et al.* 1998)] and also constructed two BAC and binary bacterial artificial chromosome (BIBAC) libraries from the cultivated cv. HA89 (Feng *et al.* 2006). Subsequently, a set of linkage group-specific BAC/BIBAC clones was identified from the libraries using the mapped cDNA-derived RFLP markers. The objectives of this study were to physically assign those linkage group-specific BAC/BIBAC clones to individual chromosomes and integrate the genetic map with the cytogenetic map in sunflower. The resulting chromosome-specific clones will be valuable cytogenetic markers for molecular cytogenetic and genomic research in sunflower. Also, the cytogenetic map will provide significant insights into a better understanding of the sunflower genomic structure and organization.

## Materials and Methods

### Plant materials

The sunflower inbred line HA89 was chosen for somatic metaphase chromosome preparation and cytogenetic map construction. HA89 has been widely used in sunflower breeding programs and also used as a parent in various mapping populations. All BAC clones used as FISH probes in the study were identified from the BAC/BIBAC libraries constructed from HA89 (Feng *et al.* 2006).

### Chromosome preparation

HA89 was planted in a greenhouse, and root tips from 2- to 3-week-old seedlings were collected and fixed in methanol: glacial acetic acid (3:1) for 1 hr. After washing in distilled water, the root tips were macerated in a mixture of 2% cellulase (Onozuka R10) and 1% pectinase (Sigma-Aldrich, St. Louis, MO) at 37° for ∼1 hr. The softened roots were carefully washed in distilled water and then fixed in the above fixation solution. One to two tips cut from roots were macerated in a drop of fixation solution using a fine-pointed forceps, and then the slide was quickly “flamed-dried” over a small alcohol lamp. The slides were examined using a phase contrast microscope and the slides with abundant well-spread metaphase chromosomes were stored at −20° for use.

### Preparation of linkage group-specific FISH probes and blocking DNA

In our previous study, 195 BAC/BIBAC clones were identified using RFLP-derived overgo probes and assigned to each linkage group (Feng *et al.* 2006) of an RFLP genetic map (Jan *et al.* 1998). These linkage group-specific clones were further used as FISH probes to align the genetic map with the cytogenetic map. A number of BAC clones for each linkage group were evaluated individually for the FISH signal to exclude clones with highly repetitive DNA sequences. Only the BAC clones that consistently produced strong and unambiguous signals were selected as chromosome-specific BAC clones for each linkage group.

BAC clone DNA was isolated by an alkaline lysate method using a QIAGEN plasmid mini kit (QIAGEN, Valencia, CA). The purified DNA was labeled with either digoxigenin-11-dUTP or biotin-16-dUTP (Enzo Life Sciences, Inc.) by nick-translation following the manufacture’s procedure (Roche Diagnostics GmbH, Germany).

Blocking DNA was prepared from HA89 genomic DNA according to Zwick *et al.* (1997) with modifications. Briefly, genomic DNA was isolated from seedlings of HA89 using the CTAB method (Rogers and Bendich 1985), and subsequently sheared by autoclaving at 100° for 1 min, followed by another cycle of 100° for 3 min. As a result, the DNA fragments, in the size range of 300−600 bp as confirmed by running a 1% agarose gel, were used as blocking DNA in the following *in situ* hybridization.

### Fluorescence *in situ* hybridization

FISH was performed according to Jiang *et al.* (1995) with minor modifications. A hybridization mixture for each slide (20 μL containing 10 μL of deionized formamide, 4 μL of 50% dextran sulfate, 2 μL of 20× saline-sodium citrate (SSC), 0.5 μL of single-stranded DNA (10 mg/mL), 1 μL of 50- to 100-ng labeled probes, and 50−100× blocking DNA (varied with probes, see Table 2) was denatured at 95° for 10 min, then immediately placed on ice for 5 min. The selected slides were denatured with 70% formamide in 2× SSC at 70° for 3 min, and then quickly dehydrated in a −20° ethanol series (70%, 90% and 100% ethanol, 5 min each). The slides were allowed to air dry at room temperature. A 20-μL drop of denatured hybridization mixture was applied to each slide and sealed with rubber cement. After overnight incubation at 37°, the cover slips were removed and the slides were washed in 2× SSC for 5 min, followed by a 30% formamide/2× SSC wash for 15 min at 42°, and then washed twice in 0.1× SSC for 8 min and in 2× SSC for 5 min at 42°.

**Table 2:** RFLP markers selected for screening the libraries and the BAC/BIBAC clones used as probes for FISH

ChromosomeNo.	SSR-LG	RFLP-LG	RFLP Marker	BAC Clone (No. Cells Examined for FISH Image)	BIBAC Clone (No. Cells Examined for FISH Image)
Ha01	1	LG12	10D6		**389J8** (64), 438D10 (12)
Ha02	2	LG14	8E4	**62M10** (14)	
Ha03	3	LG11	10C4	**60O16** (26), 150N10*^a^* (21)	
			6E6	**115B2** (22)	
Ha04	4	LG13	15E3	**67L19** (22)	402M16 (6)
			11A6		**380F19** (5), 407K6 (6)
Ha05	5	LG6	15D2	**141K9** (29), 126N9 (10), 63A12*^b^* (21)	
Ha06	6	LG4	14A2	**61N8** (31)	
Ha07	7	LG9	1C5	**60L23** (15), 183P19 (14), 184P8 (9)	
Ha08	8	LG7	7C1	**115K11** (8)	
			21E6		**429J21** (12)
Ha09	9	LG15	8C4b		**367P3***^a^*(9), 437F7 (19), 445H4 (18)
			9D1		**401C5** (5)
		LG18	13E4	**84K7** (17)	
Ha10	10	LG16	8A1	**78G18** (51)	
Ha11	11	LG17	10B5a	**110G13** (10), 159N24 (23)	368F18 (12), 428O6 (5)
Ha12^t^	12	LG5	2B4		**481K13** (18)
			2D4	**155P12** (19)	
Ha13	13	LG2	1E6		**438A20** (8)
			5E4		**387P13** (18)
Ha14^t^	14	LG1	20A5		374I4 (7), 386G6 (5)
Ha15	15	LG8	2E2	**104I23** (7)	
			20F1	**103H6** (27), 124A11 (9)	470I10*^a^* (10)
Ha16	16	LG3	9F2	**59A24** (45)	
			4B6		**464F20** (15)
Ha17	17	LG10	4D1		**381J20***^a^* (16)
			7D5	**124J4** (12), 135J2*^b^*(12)	
Total			**27**	**24** (474)	**20** (270)

Clone names in bold indicate the selected clones showing on the FISH map in Figure 2. BAC, bacterial artificial chromosome; BIBAC, bacterial /binary bacterial artificial chromosome; FISH, fluorescence *in situ* hybridization; LG, linkage group. RFLP, restriction fragment-length polymorphism; SSR, simple sequence repeats. *^t^*, numbering of the chromosomes is tentative.

a Clones showing high background when used as a FISH probe with 100× blocking DNA; all the remaining clones were applied to 50× blocking DNA.

b Clones containing pericentromeric heterochromatin sequences when used as a FISH probe.

Digoxigenin-labeled probes were detected by antidigoxigenin-rhodamine (Roche Diagnostic, Indianapolis, IN), and biotin-labeled probes were detected by fluorescein avidin DCS (Vector Laboratory, Inc., Burlingame, CA). The slides were counterstained with 4′,6-diamidino-2-phenylindole (DAPI). Fluorescence signals from different probes were captured individually with appropriate filters under a Zeiss Axioplan 2 epifluorescence microscope (Carl Zeiss Light Microscopy, Jena, Germany), and then merged using Axiocam & Axiovision 3.1 software (Carl Zeiss Light Microscopy).

### Coalescence of an independently developed RFLP map with SSR genetic maps

In the previous research, Yu *et al.* (2003) and Gedil *et al.* (2001) partially integrated the RFLP genetic map of Jan *et al.* (1998) with the public SSR genetic map of Tang *et al.* (2002). Of 17 linkage groups, 13 linkage groups were cross-referenced by a total of 40 shared RFLP marker loci (Jan *et al.* 1998). Four linkage groups (LG), LG1, LG4, LG5, and LG8 of the RFLP genetic map (the prefix “LG” refers to a linkage group from Jan's RFLP map; see Table 2), have not yet been unified into the public SSR genetic map (Gedil *et al.* 2001; Yu *et al.* 2003). To further integrate the remaining four linkage groups, 17 RFLP-based sequence-tagged sites (STS) markers from LG1 (2 markers), LG4 (6 markers), LG5 (6 markers), and LG8 (3 markers) (Jan *et al.* 1998) and 49 SSR markers from SSR-LG6 (10 markers), SSR-LG12 (13 markers), SSR-LG14 (11 markers), and SSR-LG15 (14 markers) [“SSR-LG” refers to a linkage group from the SSR genetic map (Tang *et al.* 2002; Yu *et al.* 2003)] were genotyped with 96 F_2_ plants from a cross of CMS HA89×RHA280. Genomic DNA was extracted according to the protocol of the QIAGEN DNAeasy 96 Plant Kit (QIAGEN). The polymerase chain reaction amplification and gel electrophoresis followed Liu *et al.* (2012). A partial linkage map was constructed using the software MAPMAKER/Exp version 3.0b [Whitehead Institute, Cambridge, MA (Lander *et al.* 1987)], and linkage group maps were drawn using Mapdraw V2.1 (Liu and Meng 2003).

## Results

### Development of chromosome-specific markers

We previously identified 195 BAC and BIBAC clones from HA89 genomic DNA libraries using 36 single or low-copy RFLP-derived overgo probes (Feng *et al.* 2006). Each RFLP marker hit multiple BAC/BIBAC clones. The plasmid DNA isolated from these BAC/BIBAC clones could be used as DNA probes for FISH mapping. However, due to the large genome of sunflower with abundant repetitive sequences, many of these BAC/BIBAC clones (∼100 kb in size) contain a large proportion of repetitive DNA sequences that might produce dispersed repetitive signals along the chromosomes when used as FISH probes. Therefore, *in situ* hybridization with those BAC/BIBAC clones required the inclusion of blocking DNA in the hybridization mixture to eliminate background noise. In our initial screening experiments, only a few BAC/BIBAC clones gave clear signals without blocking DNA. Most of the BAC/BIBACs resulted in moderate background even with blocking DNA. Therefore, in this study, all the probes were applied with 50× blocking DNA to minimize the cross-hybridization of the repetitive DNA sequences. However, some of the clones produced a relatively high background even with 50× blocking DNA. In this case, 100× blocking DNA was applied to these clones (Table 2).

**Figure 1:**
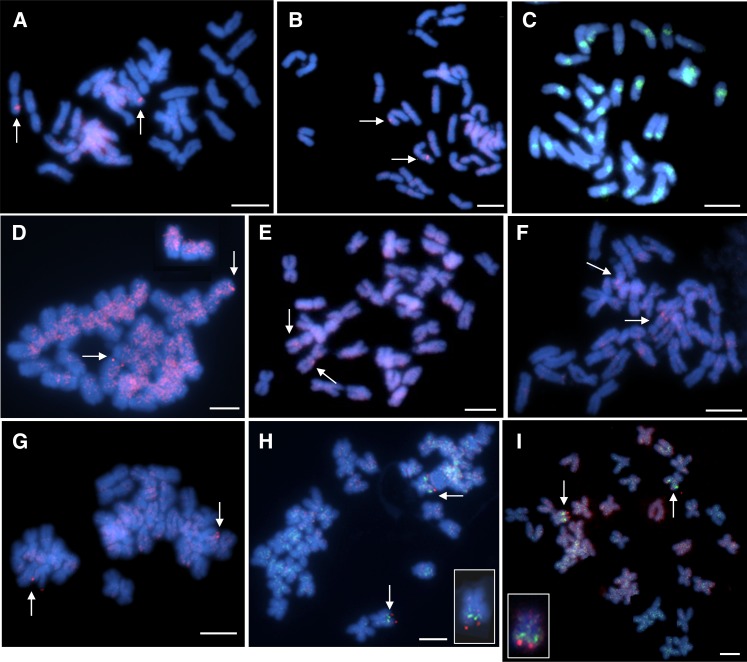
An entire metaphase cell with 34 chromosomes counterstained with DAPI (blue). (A, B) FISH of a BAC clone 59A24 generated a specific signal (arrow) on chromosome Ha16; (C) FISH image of BAC clone 135J2 corresponding to RFLP marker 7D5 on Ha17 showing a strong dispersed pattern on the pericentromeric region of almost all sunflower chromosomes, and reflecting the distribution of repetitive DNA; (D) With 100× blocking DNA applied, clones 470I10 (Ha15) produced hybridization signals (arrow) on one pair of chromosome with high background due to nonspecific binding of the probe; (E-F) Two BAC clones, 401C5 (E) and 367P3 (F), identified with RFLP markers from same linkage group LG15, were FISH-mapped on two different morphologic chromosomes based on two independent *in situ* hybridizations. The clone 401C5 was localized on a metacentric chromosome and 367P3 on a satellite chromosome. (G) 84K7 from LG18 was located on one submetacentric chromosome; (H, I) BAC clones 367P3 from LG15 and 84K7 from LG18 were cohybridized on one submetacentric chromosome Ha09. 367P3 (green) was located proximal to the centromere and 84K4 (red) distal to the centromere. Insets in H and I: magnified images for two chromosomes showing two-color FISH with two probes. The bars indicate 5 μm.

**Figure 2:**
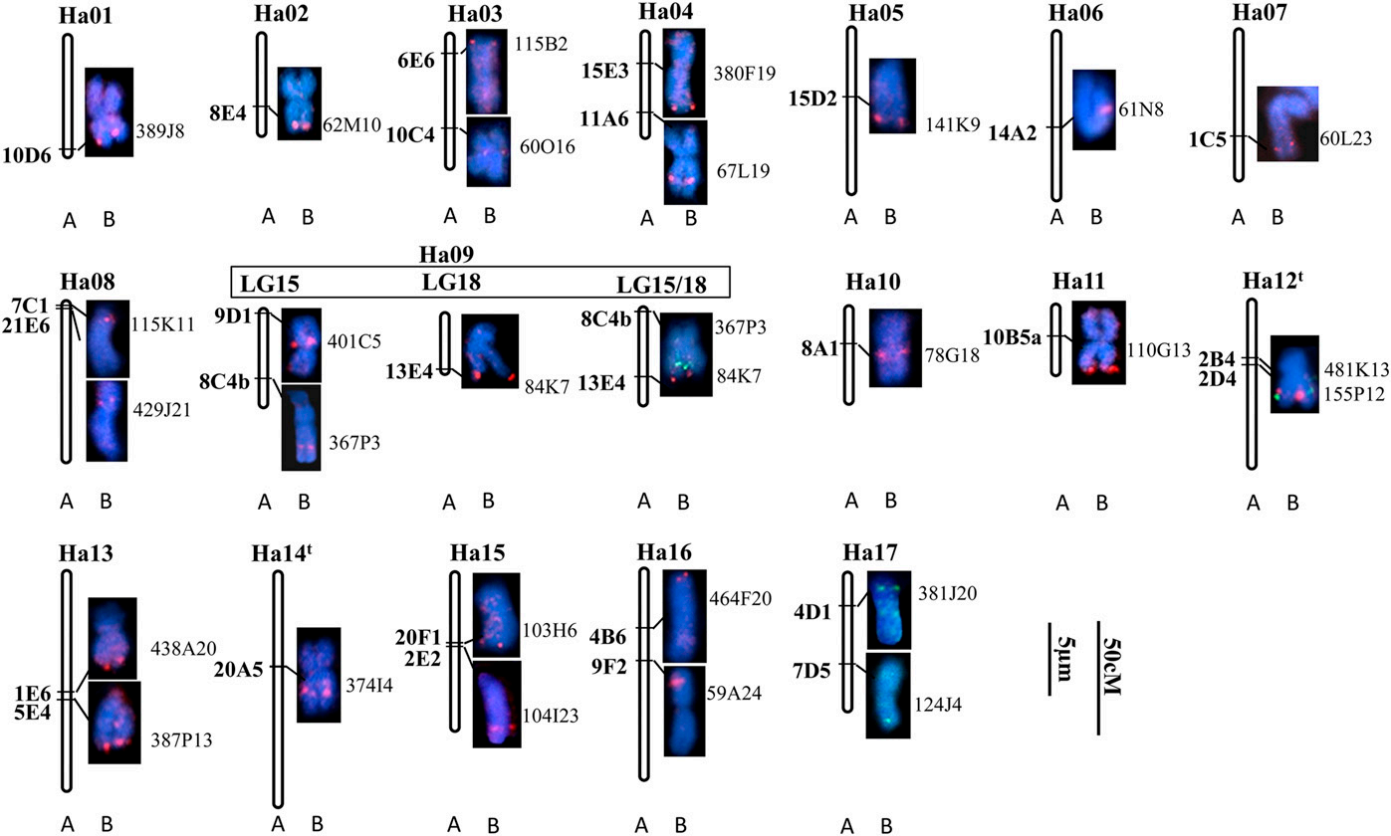
Identification of individual chromosomes and comparison with the linkage map (Jan *et al.* 1998). The RFLP genetic map with RFLP markers (A) was used to select BAC clones to identify their corresponding chromosomes (B) by FISH. Numbering of the chromosomes follows Tang *et al.* (2002). Ha12^t^ and Ha14^t^ indicate numbering of the chromosomes is tentative. The RFLP markers are showed on the left of each chromosome in bold, and the names of corresponding BAC clones are on the right. The red signals were digoxigenin-labeled probes detected by antidigoxigenin-rhodamine, and the green signals were biotin-labeled probes detected by avidin-fluorescein. All slides were counterstained with DAPI (blue). The bars indicate 5 μm for chromosomes and 50 cM for the linkage maps. Note that the length of chromosomes cannot be compared in the figure as they were derived from different cells.

One to four BAC/BIBAC clones for each linkage group were selected as probes to perform FISH in the present study. Each slide hybridized with an individual probe was thoroughly examined under a fluorescence microscope, and the FISH results were analyzed and summarized in Table 2. The true hybridization signals, which consistently appeared at the same chromosomal locations in the majority of cells examined (Figure 1, A and B), were deemed to be chromosome-specific and considered for analysis. Of the 61 BAC/BIBAC clones tested, 16 clones (∼26%) either could not detect a distinctive signal on a specific chromosome, such as multi-loci; or generated a high background with dispersed signals along most of chromosomes. These clones were excluded from the FISH experiments. The remaining 44 clones (24 BACs, 20 BIBACs) that encompass 18 RFLP linkage groups (for clarity, the prefix “LG” refers to a linkage group from Jan’s RFLP map; see Table 2) were selected for FISH analysis in this study (Table 2). The smallest linkage groups, *i.e.*, LG19 and LG20, were not included in this study because there were no sequenced RFLP markers available (Jan *et al.* 1998). Some of the clones, such as 135J2 for the RFLP marker 7D5 on LG10, produced specific signals on the pericentromeric heterochromatin of almost all sunflower chromosomes (Figure 1C). In addition, clones with rDNA-like repetitive sequence were also found. These clones are useful for the analysis of DNA sequences in the nucleolus organizing region or centromeric region. As stated previously, HA89 blocking DNA (50×) was applied to all the hybridization reactions. However, some of the clones such as 470I10 (LG8) still produced signals at more than one site, either due to homology or nonspecific binding of the probe. Under this circumstance, we differentiated the primary hybridization site from minor ones. Also, we increased the blocking DNA to 100× to minimize the cross-hybridization for these probes (Figure 1D). However, the most effective way for BAC-FISH is still to select BAC/BIBAC clones containing relatively small segments of dispersed repetitive sequences and large segments of unique sequences that would generate unambiguous FISH signals (Figure 1, A and B). Based on the relative signal strengths between the true target and cross-hybridization sites, each of the 27 BAC/BIBAC clones was anchored to one of the 18 linkage groups. They can be used as chromosome-specific cytogenetic markers for the sunflower genome (Figure 2).

### Establishment of putative nomenclature for sunflower chromosomes

**Figure 3:**
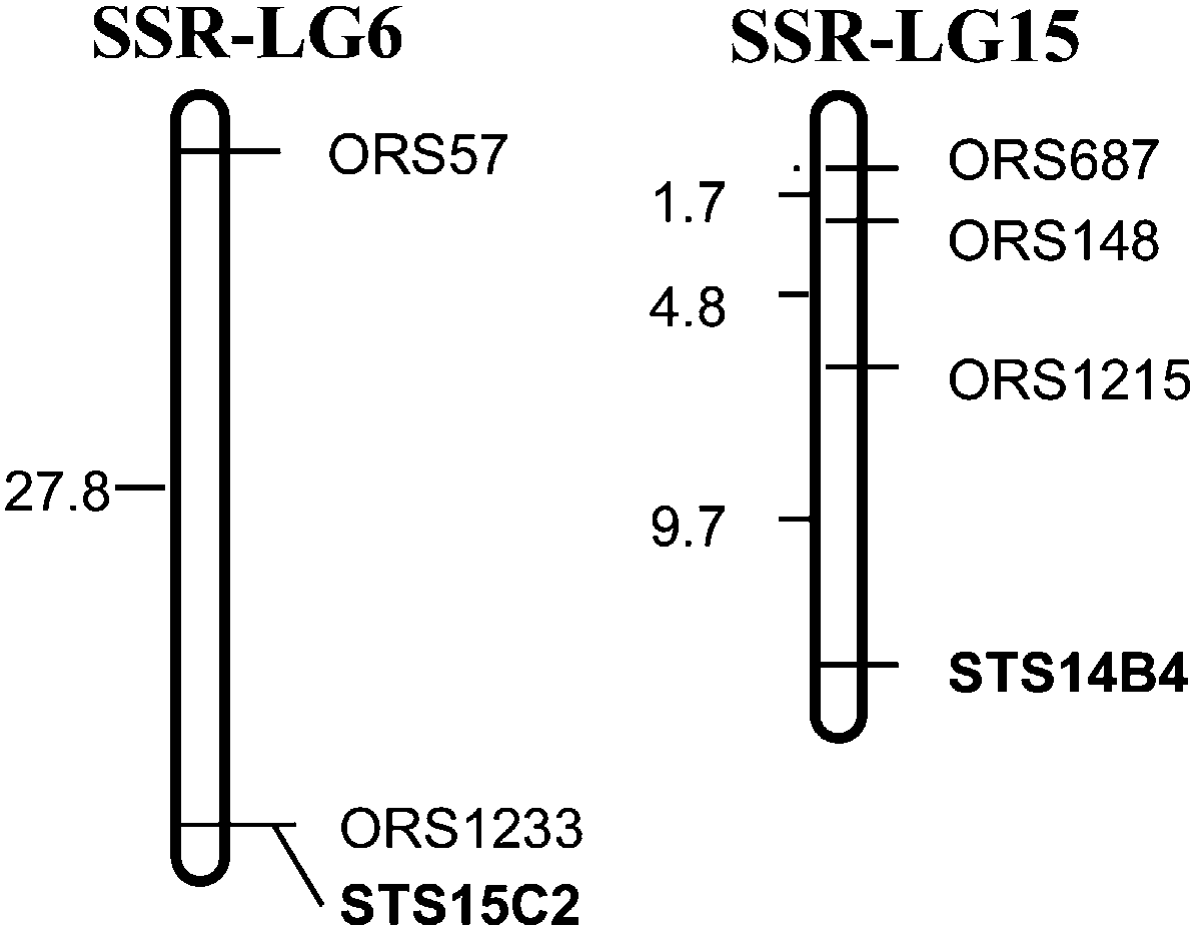
Partial linkage map of SSR-LG 6 and SSR-LG15 (Tang *et al.* 2002) with two linked RFLP markers (Jan *et al.* 1998), based on the analysis of an F_2_ population from the cross of CMS HA89 x RHA280. Markers labeled “STS” were RFLP-derived markers. Markers labeled “ORS” were SSR markers. The distances are in cM.

The public SSR genetic map with 1089 SSR markers as a genome-wide framework for sunflower genetic analysis has been integrated with a number of other genetic linkage maps (Berry *et al.* 1997; Jan *et al.* 1998; Gedil *et al.* 2001; Paniego *et al.* 2002; Tang *et al.* 2002; Al-Chaarani *et al.* 2004) and has been widely used as the public reference map by sunflower research community. Henceforth, in the present study, we propose a chromosome nomenclature and designate sunflower chromosomes according to the corresponding SSR linkage groups from 1 to 17 (Tang *et al.* 2002; Yu *et al.* 2003) with the “Ha” prefix, indicating cultivated sunflower *Helianthus annuus* species. Previous reports have integrated 13 linkage groups between the public SSR map and Jan’s RFLP map, and four linkage groups remain unlinked (Gedil *et al.* 2001; Yu *et al.* 2003). To further unify those four linkage groups, 17 RFLP and 49 SSR markers from the four linkage groups were selected to screen polymorphic between the parents of a cross CMS HA89 × RHA280, resulting in four RFLP and 25 SSR markers showing polymorphic. Subsequently, all polymorphic markers were used to genotype the F_2_ population. The results showed that RFLP marker STS15C2 from LG4 of Jan’s genetic map co-segregated with ORS1233 and was loosely linked with ORS57 from SSR-LG6; in addition, RFLP marker STS14B4 from LG8 of Jan’s genetic map was linked to ORS1215, ORS148, and ORS687 from SSR-LG15 (Figure 3). The results suggested that LG4 and LG8 of Jan’s RFLP map were aligned with SSR-LG6 and SSR-LG15, respectively. The other two RFLP linkage groups could not be coalesced into the SSR genetic map due to a limited number of polymorphic RFLP markers available. However, one RFLP marker, 20A5 on LG1, showed a loose link (under LOD 2.0) to ORS580 on SSR-LG14 (data not shown). So, tentatively, we proposed that LG1 of Jan’s RFLP map was associated with SSR-LG14. At this point, we assumed that the last one, LG5 of Jan’s RFLP map, might be associated with SSR-LG12. Nevertheless, the process of cross referencing the two maps is still going on by designing more markers for those linkage groups and genotyping other mapping populations. We are confident that all the RFLP linkage groups of Jan’s map will eventually integrate into the public SSR genetic map when further linked loci are located. Based on all current data, we proposed a putative chromosome nomenclature system to facilitate the development of cytogenetic map of sunflower. The correspondence between the current chromosome nomenclature and SSR linkage groups, as well as with Jan’s RFLP linkage groups is presented in Table 2.

### Assignment of RFLP linkage groups to individual chromosomes

Clones bearing unique sequences enabled the alignment of genetic markers onto chromosomes. A total of 27 BAC/BIBAC clones were assigned to 17 sunflower chromosomes and designated according to the corresponding SSR linkage groups of Tang *et al.* (2002), naming Ha01 to Ha17 (with exception of Ha12^t^ and Ha14^t^, indicating tentatively; Table 2, Figure 2). Among them, chromosomes of Ha03, 04, 08, 09, 12^t^, 13, 15, 16, and 17 were associated with two BAC/BIBAC clones; the remaining chromosomes with one clone each (Figure 2). Some clones, including 60O6 (Ha03), 67L19 (Ha04), 61N8 (Ha06), 429J21 (Ha08), 401C5 (Ha09), 78G18 (Ha10), and 374I4 (Ha14^t^) were located interstitial of the arms or close to the centromeres. However, the majority of the clones, such as 62M10 (Ha02), 380F19 (Ha04), 84K7 (Ha09), 110G13 (Ha11), 438A20 and 387P13 (Ha13), and 464F20 (Ha16) were located near the ends of the chromosome arms. This finding is consistent with the fact that the distal regions of a chromosome are euchromatin-rich, characterized by a relatively high density of genes. In other words, there was good correspondence of the FISH hybridization sites on the chromosome with the respective marker location on the genetic linkage map. These results allowed for the assignment of each linkage group to an individual chromosome in cultivated sunflower. Based on the chromosome size and morphology, sunflower chromosomes can be placed into three groups, *i.e.*, submetacentric satellite chromosomes, acrocentric chromosomes, and metacentric/submetacentric chromosomes. The following are the assignments of the genetic linkage groups onto individual chromosomes in these three groups.

### Submetacentric satellite chromosomes

This group includes the most distinctive satellite chromosomes corresponding to LG2 (Ha13), LG5 (Ha12^t^), LG18 (Ha09), and LG15 (Ha09) of Jan’s RFLP genetic map. FISH using probes 438A20 and 387P13 produced distinct signals at the end of the long arm of chromosomes Ha13. Bicolor FISH simultaneously determined the physical localizations of two BACs, 481K13 (red) and 155P12 (green), which correspond to RFLP markers 2B4 and 2D4, respectively. Both clones were clearly localized near the end of the long arms of chromosome Ha12^t^. BAC clone 481K13 (red) resided proximal and 155P12 (green) distal to the centromere (Figure 2). These results provided significant information for chromosome identification. Two BAC clones, 401C5 and 367P3 identified with RFLP markers from LG15, were FISH-mapped onto two different chromosomes based on two independent *in situ* hybridizations (Figure 1, E and F). Clone 367P3 was localized in the interstitial region of the long arm on a satellite chromosome, whereas 401C5 was close to the centromere on a metacentric chromosome. The probable reason for this will be discussed later. Another satellite chromosome corresponding to LG18 showed a strong signal at the very end of the long arm when using probe 84K7 (Figures 1G and 2).

It is apparent that some large gaps remain in the RFLP genetic map by Jan *et al.* (1998), which split a large linkage group into small ones, such as LG17, 18, 19 and 20. These small sublinkage groups will be eventually assembled as more molecular markers are added to the genetic map, or more BAC clones are applied to a cytogenetic map. According to the composite SSR reference map, two markers located on LG15 (9E6) and LG18 (14D2) in Jan’s RFLP genetic map were assigned to SSR-LG9 (Yu *et al.* 2003). To determine whether these RFLP marker loci belong to the same linkage group, BAC clones 367P3 from LG15 and 84K7 from LG18 were cohybridized to one mitotic metaphase spread. The resulting FISH signals from two probes were found to colocalize on one submetacentric chromosome Ha09. BAC clones 367P3 (green) localized proximal to the centromere and 84K7 (red) distal to the centromere (Figures 1, H and I, and 2). With LG15 and LG18 belonging the same linkage group, it can be concluded that four RFLP linkage groups, LG2, LG5, LG15, and LG18, correspond to three chromosomes, Ha13, Ha12^t^, and Ha09, bearing nucleolus organizing regions. This finding is consistent with our previous report that three pairs of chromosomes showed distinctive FISH signals when using 45s rDNA as probes (Feng *et al.* 2005). In addition, the aforementioned result not only provided important information for integration of two small linkage groups but also provided supporting evidence for LG15 mentioned previously. Clones 401C5 and 367P3 from LG15, corresponding to Ha09, were assigned to two morphologically different chromosomes. Clone 401C5 was localized on a metacentric chromosome and 367P3 on a satellite chromosome (Figure 1, E and F). This may suggest that RFLP markers 9D1 (401C5) and 8C4b (367P3) (Figure 2) probably do not share the same linkage group. Further experiments are underway to verify the chromosomal locations of the BAC clones assigned to the same LG.

### Acrocentric chromosomes

Three chromosome pairs, Ha05, Ha07, and Ha08, were characterized by a small short arm or lack of a visible short arm and called acrocentric or subtelomeric chromosomes. The BAC clone 141K9 generated a clear signal on the long arm of Ha05. The chromosome Ha07, a large acrocentric chromosome with a visible short arm, was unambiguously hybridized by 60L23, which corresponded to RFLP marker 1C5. For the chromosome Ha08, two BAC clones, 115K11 and 429J21, were localized near the centromeric region with 115K11 proximal and 429J21 distal to the centromere. This physical location concurred with their positions on the linkage map.

### Metacentric/submetacentric chromosomes

All other chromosomes in sunflower were identified as metacentric/submetacentric characterized by the similar length of the two arms. They included the chromosomes Ha01, 02, 03, 04, 06, 10, 11, 14^t^, 15, 16, and 17. Of them, Ha01, 04, 06, 11, 14^t^, and 16 are large chromosomes, and the others are of intermediate size.

Ha01, a large metacentric chromosome, was identified by a clear FISH signal generated by clone 389J8 near the end of the short arms. Another large chromosome Ha04 was hybridized by two clones, 380F19 generating a signal at the end of the long arms, and 67L19 corresponding to an interstitial signal. For Ha06, the BAC clone 61N8 showed strong interstitial signals on the short arms in 31 cells examined. Chromosome Ha11 corresponding to LG17 with a short genetic distance due to limited RFLP marker available, was associated with BAC clone 110G13 at the end of chromosome arms. The chromosome Ha14^t^, a large metacentric chromosome, was clearly hybridized with BAC 374I4 in the middle of the long arms. This is highly consistent with the genetic location of the marker on the RFLP linkage map. On chromosome Ha16, the BAC clone 464F20 generated the FISH signals near the end of the chromosome arms, and clone 59A24 identified by the RFLP marker 9F2 showed a strong interstitial signal on the short arm (Figures 1, A and B, and 2).

For the intermediate size chromosomes, Ha02 was hybridized by BAC clone 62M10 at the end of chromosome arms. Ha03 was identified by two clones 115B2 and 60O16. BAC clone 78G18 presented a clear FISH signal very close to the centromere on chromosome Ha10. Ha15 was hybridized by two clones, 103H6 at the end of the chromosome arms, and 104I23 in the interstitial region of the chromosome arms. Finally, Ha17 was characterized by two clones 381J20 and 124J4 at the end of the chromosome arms (Figure 2).

Taken together, 44 BAC/BIBAC clones corresponding to 27 RFLP markers were applied to mitotic metaphase spreads using a one- or two-color detection system. Most of the BAC/BIBACs yielded a clear single FISH signal on chromosome pairs. Of these, 27 BAC/BIBACs generating specific signals were placed on the cytogenetic map (Figure 2). This allowed us to assign each genetic linkage group to a specific sunflower chromosome.

## Discussion

Successful establishment of sunflower cytogenetic map in the present study largely attributed to a series of our comprehensive research. Previously, we developed a sunflower RFLP linkage map with 20 linkage groups using cDNA probes (Jan *et al.* 1998); and constructed two BAC and BIBAC libraries from the cultivated sunflower (Feng *et al.* 2006). Subsequently, a set of linkage group-specific BAC/BIBAC clones was identified from the libraries using the mapped cDNA-derived RFLP markers. Using these linkage group-specific BAC/BIBAC clones as probes, 27 BAC/BIBAC clones were assigned to individual sunflower chromosome corresponding to their genetic linkage group. This demonstrates the potential of large-insert DNA libraries for the development of molecular cytogenetic resources for sunflower, and allows for the assignment of a genomic clone to a specific chromosome without the need for chromosome identification by banding patterns.

### Quality chromosome preparation and single- or low-copy genomic sequences are essential

The successful application of BAC-FISH technique depends on the quality of the chromosome spread and the percentage of repetitive DNA sequences contained in the selected probes. The sunflower genome contains a high percentage of repetitive DNA sequences, making it difficult to develop chromosome-specific FISH probes. The most effective way to generate unambiguous FISH signals is to select BAC/BIBAC clones containing relatively few dispersed repetitive sequences and high proportion of unique sequences. With various sequences of genomic DNA, cDNA, and mRNA available, one strategy to develop FISH probes without the interference of repetitive sequences is to pool multiple PCR products with low-copy sequences from BACs (Lamb *et al.* 2007).

BAC-FISH techniques have not been widely used in sunflower mainly due to difficulties in chromosome preparations (Paniego *et al.* 2007). Signal sensitivity of *in situ* hybridization largely relies on the use of high-quality metaphase preparations with well-spread chromosomes that are free from cytoplasm. So far, in sunflower, *in situ* hybridization has mainly been reported for rDNA probes with high repetitive sequences or other repetitive retrotransposon-like sequences (Talia *et al.* 2010), which produce much stronger signals than single copy sequences. For single-copy BAC/BIBAC clone probes, quality chromosome preparations are essential. Based on our observations, compared with wheat, barley, and maize with relatively larger chromosomes, sunflower possesses smaller chromosomes and high-density cytoplasm. The signal intensity is largely affected by the amount of cytoplasm adhering to the chromosomes, as well as by the physical position of the chromosome itself (*i.e.*, how flat the chromosome may be on the slide). In addition, in some species like rice, FISH mapping was conducted on pachytene chromosomes. But in sunflower, identification of individual chromosomes on a meiotic spread can be a problem because of the large number of chromosomes and high-density cytoplasm. Therefore, in the present study, unlike universal “squashing” in 45% acetic acid, we used a “flamed-dried” method to prepare somatic chromosomes to eliminate the dense cytoplasm and allow chromosomes to be well spread on glass slides.

### Regarding sunflower nomenclature system

The early cytological research on sunflower mainly focused on chromosome classification and karyotype analysis. Seventeen chromosome pairs of cultivated sunflower were classified into metacentric, submetacentric, and subtelocentric groups, numbering 1 to 17 based on the chromosome length and arm ratios. It is obvious that chromosome numbering among different reports was inconsistent and cannot be cross-referenced even for the same variety (Table 1). For example, chromosomes 1 to 4 of HA89 karyotype were depicted as metacentric by Cuellar *et al.* (1996); however, Schrader *et al.* (1997) defined them as acrocentric. Similar problems existed in other plant species or varieties. The question raised here is how to establish a sunflower chromosome nomenclature system, or how to associate linkage groups with chromosomes.

In general, karyotype analysis relies on the arm ratio, chromosome size, heterochromatic elements, or rDNA distribution in the metaphase stage of root-tip cells, and this size-based nomenclature was adopted for most plant species. However, the sunflower chromosomes are relatively small compared with wheat, barley, and even maize. Chromosome sizes vary widely due to the different contraction of the chromosomes measured at different stages. Also, chromosome morphology might vary among varieties and accessions due to chromosome rearrangements during evolution. Therefore, designating a chromosome only based on its morphology and banding pattern may not be reliable and frequently requires revision or reassignment (Pedrosa-Harand *et al.* 2008). In the current chromosome-specific marker system, we designated sunflower chromosomes according to their corresponding SSR genetic linkage groups (Tang *et al.* 2002, Yu *et al.* 2003), which have been widely accepted by the sunflower community. In addition, Jan’s RFLP linkage groups, from which the current FISH probes were developed, have been integrated and cross-referenced with the public SSR genetic map (Tang *et al.* 2002; Yu *et al.* 2003). Eventually, with the technical improvement and knowledge enrichment for sunflower cytogenetics, a uniform and standard nomenclature can be proposed and adopted to facilitate scientific communication for the sunflower crop. To some extent, this work provides a significant platform for sunflower cytogenetic research.

### Confirm ambiguous RFLP linkage group and integrate cytogenetic map and genetic map in sunflower

It is notable that the RFLP genetic linkage map by Jan *et al.* (1998) included 20 linkage groups, which is three more than the 17 haploid chromosome number of cultivated sunflower. Four small linkage groups, LG17, LG18, LG19, and LG20, must have been split from large groups due to insufficient marker data, as evidenced by the presence of several gaps along the linkage map. Accordingly, by using a dual-color FISH technique, two BAC clones selected from RFLP LG15 and LG18 generated clear signals on the same chromosome (Figure 1, H and I). In addition, both chromosomes were identified as subtelocentric chromosomes from two independent *in situ* hybridizations when using their corresponding single BAC clone as probe. Therefore, the FISH signals and chromosome morphology suggest that the chromosomes corresponding to LG15 and LG18 of the RFLP genetic map of Jan *et al.* (1998) are the same. This result provides significant physical information for the previous genetic map, in which small linkage groups can be coalesced together when additional linked loci are identified. Also, based on the map of Yu *et al.* (2003), two RFLP markers UB5E6 (LG12) and UB6F2 (LG17) of Jan’s map were co-located on the same linkage group SSR-LG11 of Yu *et al.* (2003). Similar report was found in Gedil *et al.* (2001). Therefore, there is no doubt that the BAC-FISH technique developed currently will continue to play a substantial role in integration of sunflower genetic maps with the physical map, and also rectify the previous linkage maps.

### Recombination activity

A few RFLP markers were defined at proximal positions within their corresponding linkage groups; however most BACs were localized to the distal regions of chromosomes. Of the 27 BAC probes on the cytogenetic map, 18 clones were localized to or near the end of chromosome arms. These results are consistent with reports of strongly reduced levels of recombination in the proximal region of each chromosome (Heslop-Harrison 1991). Similar results were also reported in rice genome (Wu *et al.* 2003), in which they found that there were marked changes in the relative recombination rate along the length of each chromosome, and chromosomal recombination at the centromere core and surrounding regions on the six chromosomes investigated was completely suppressed. Nakamura *et al.* (1997) FISH-mapped two BAC clones closely linked to the rice blast resistance gene Pi-b at 96.2% distance from the end of the short arm of chromosome 2 and Pi-ta2 in the centromeric region of chromosome 12. Their results also showed that high frequency of repetitive sequences near centromere may contribute to the low recombination rate in the region. Genomic sequence analysis of these regions should provide more details to understand the mechanism of activation and inactivation of recombination frequency along the chromosome. A physical map of a chromosome is significant and informative for genomic research, since it allows researchers to determine the location of genes, unanchored BACs, and other genomic organization and structure.

In sunflower, although significant challenges still remain, such as the simultaneous hybridization on the same cell by multi-probes, this research will facilitate the establishment of a universal sunflower physical map and chromosome nomenclature system. As chromosome-specific cytogenetic markers, the selected BAC/BIBAC clones that encompass the 17 linkage groups provide a valuable tool for identifying sunflower cytogenetic stocks and tracking alien chromosomes in interspecific cross progenies. A set of sunflower trisomics has been developed, and we are currently identifying each extra chromosome by using chromosome-specific cytogenetic markers. In addition, the established techniques enable researchers to assign more BAC clones and to develop a more informative cytogenetic map. This study lays the groundwork not only for integrating the genetic map with the physical map of sunflower, but also for resolving problems in previous linkage maps.
